# Why the National Institutes of Health should create an institute of positive biology

**DOI:** 10.1258/jrsm.2012.120113

**Published:** 2012-10

**Authors:** Colin Farrelly

**Affiliations:** Queen's University – Political Studies, Kingston, Ontario, K7L 3N6, Canada

The National Institutes of Health (NIH) is the largest source of funding for medical research in the world. With an annual budget currently over $31 billion, the NIH awards more than 50,000 competitive grants to thousands of researchers working in the USA and around the world. The NIH is composed of 27 distinct institutes and centers, many of which are institutes for specific diseases, such as the National Cancer Institute (NCI), the National Institute on Alcohol Abuse and Alcoholism (NIAAA), the National Institute of Allergy and Infectious Diseases (NIAID) and the National Institute of Neurological Disorders and Stroke (NINDS), just to name a few.

With so many institutes dedicated to the study of specific diseases and disorders, one might draw the conclusion that *well-ordered* science and medicine mandates that the bulk of our energies and resources be invested in trying to answer the fundamental question ‘what causes pathology?’. Elsewhere^1^ I have called this intellectual presupposition the paradigm of ‘negative biology’, and this paradigm dominates the medical sciences, including the design of the NIH. In this essay, I argue that we need to supplement the disease-specific approach of negative biology with an ambitious commitment to the study of what can be called ‘positive biology’. Rather than focusing on understanding the proximate causes of disease, positive biology seeks instead to understand the proximate and evolutionary causes of *exemplary* positive phenotypes – such as exceptional healthy ageing, play and happiness, resilience, and optimism. To help make the aspirations of positive biology both coherent and concrete, I detail how the creation of a new NIH Institute – the Institute of Positive Biology (or IPB) – could help promote the health, happiness and prosperity of today's populations.

## Why the current focus on pathology?

The lion's share of the public funding for health research the NIH allocates is invested in the study of pathology rather than the study of ‘health’ *per se*. The vast majority of the 233 listed research topics on the NIH's ‘Estimates of Funding for Various Research, Condition, and Disease Categories (RCDC)’ involve research into specific diseases.^2^ The estimated investment, for the year 2013, in disease research includes $5.4 billion for cancer, $3.9 billion for infectious disease, $3.9 billion for brain disorders, $3.5 billion for rare diseases, $3 billion for HIV/AIDS and $2 billion for cardiovascular disease. Some funded research areas are not explicitly disease focused. These include women's health ($3.9 billion), ageing ($2.6 billion), nutrition ($1.4 billion) and health disparities ($2.7). Yet even within these areas of research the focus on pathology research is significant. The creation of a new Institute of Positive Biology could help expand the NIH's focus beyond the confines of negative biology.

Why is medical research dominated by the study of pathology? A *complete* explanation would no doubt identify many distinct factors, but I believe that two important factors are worth noting here which can help us appreciate why we need to go beyond the aspirations of negative biology. The first factor is that humans are susceptible to ‘observation biases’. We are more likely to observe human tragedy and suffering than we are human health and happiness. When driving we slow down to closely observe car accidents, but we seldom observe the fact that most drivers on the road avoid accidents and safely arrive at their destinations. The image of the carnage of a car accident can have a moving and lasting impression upon our psyche, the same is not true of the observation of the *absence* of accidents.

The same cognitive bias occurs in our thinking about human health and our biology. It is natural for us to want to harness science to cure, treat and prevent specific diseases because these diseases are what kill most humans living in the world today. The World Health Organization estimates that, in the decade between 2005 and 2015, 76 million people will die from chronic illness in high-income countries.^3^ The number of deaths in this decade is even higher for the more populous lower-middle-income countries, such as China and India. It is estimated that chronic illness will cause 144 million deaths in these lower-middle-income countries.^4^ In light of such staggering statistics, it is easy to understand why we assume it is most prudent to invest our time, energy and resources into pathology research. If we better understand the genetic and environmental factors at play with cancer, we may be able to reduce, treat or perhaps even eliminate cancer as a cause of death.

## Moving beyond the focus on pathology

**Figure 1:**
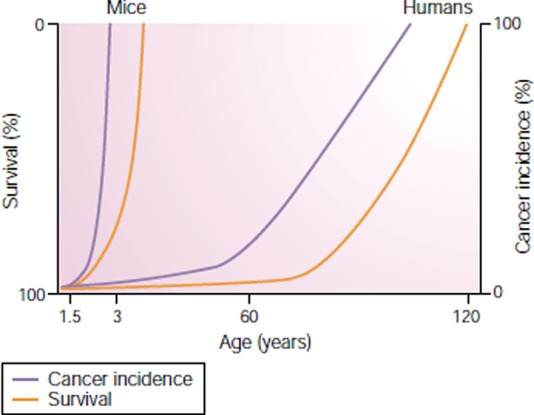
Cancer increases, for mice and humans, with ageing.**^5^** (Reprinted with permission from Macmillan Publishers Ltd: *Nat Rev Genetics*, copyright [2003])

The disease-specific approach to health research presumes that a ‘disease-free’ state of health and longevity is the norm, and what needs to be explained are deviations from this ideal. Comparative biology demonstrates that this is not the case. A comparison of cancer mortality in humans and mice (Figure 1) effectively illustrates the reality that ageing leaves mammals vulnerable to disease. The development of disease in late life is thus ‘normal’.

While the reasons humans develop disease in late-life is a puzzle worth seriously contemplating, an even more fascinating and pressing puzzle is figuring out how some (rare) individuals can live a century of disease-free life. In the USA and other industrialized countries, it is estimated that the prevalence rate of centenarians is about one in 6000 people.^6^ Some of these centenarians are ‘delayers’, which means they delayed the onset of age-associated illness. A second category of centenarians are survivors. These are persons who were diagnosed with an illness prior to age 80, but survived for at least two more decades. And the third category of centenarians are ‘escapers’, people who escaped the most lethal diseases, such as heart disease, non-skin cancer and stroke.^7^ Empirical studies suggest that there is a significant genetic component to the ‘healthy ageing’ experienced by centenarians. Futhermore, a compression of morbidity has been observed in supercentenarians (age 110–119 years).^8^ The development of a novel medical intervention which would permit us to replicate the biology of centenarians or supercentenarians in the average person would be among this century's greatest medical breakthroughs. But such research is marginalized by the current disease-model approach to health extension which the NIH has adopted (Table 1).

**Table 1:** Contrast between negative and positive biology

**Starting intellectual assumptions**
*Negative biology*: Health, longevity and happiness are assumed to be a ‘given’, or part of ‘normal species functioning’, for humans.
* Positive biology*: There is diverse variation in the genotypes which influence desired phenotypes, such as health. The evolutionary and life history of different species helps explain this variation and the different biological tradeoffs that determine age of reproduction, body size, senescence, complexity of the brain, etc.
**What needs to be explained?**
* Negative biology*: The proximate causes of disease, frailty and disability.
* Positive biology*: The proximate and ultimate causes of exceptional health, positive emotions and happiness, high cognitive ability, etc.
**Which kinds of interventions ought to be pursued?**
* Negative biology*: Interventions that help prevent, treat and cure *specific* diseases.
* Positive biology*: Interventions that increase the opportunities for health, happiness and wellbeing.

While it is true that the National Institute on Aging (NIA) includes the ‘Biology of Aging Program: Understanding Aging Processes, Health, and Longevity’, this program receives only a small fraction of the funding the NIA allocates towards the study of specific diseases of ageing. The President's requested budget for the Biology of Aging Program in 2013 is $176.251 million, and yet the amount requested for Alzheimer's disease (AD) alone is $271.5 million.^9^ So AD, which is only one specific disease of ageing, receives nearly $100 million more in public funding than the total budget invested in understanding the biology of ageing. The creation of the IPB would help ensure that the study of healthy ageing is able to stand on a more equal footing with research on pathology. Under the current pathology-focused model, the benefits of positive biology have little to no chance of ever being realized.

A second reason we have invested so much in the focus on specific diseases is that this approach reaped enormous health dividends in the 20th century, by reducing early and mid-life mortality. Over the course of that century, life-expectancy in the USA rose from 49 to 77 years.^10^ This dramatic rise was caused by many factors – such as technological advances, increased material prosperity, changes in behaviour (e.g. birth control), etc. – but the most significant medical advances arose from the application of insights from epidemiology's focus on the proximate causes of disease. This led to significant public health measures, like the sanitation revolution, antibiotics and the small pox vaccine. Focusing on pathology made a great deal of sense when the main cause of death was communicable disease. But the health challenges facing today's ageing populations are different from the health challenges which populations faced in the early 20th century. Expanding health in late life is far more difficult because of the reality of co-morbidity.^11^ The fixation on research for specific diseases has not resulted in the elimination of a single chronic disease. The pathology-focused strategy has helped increase the amount of time individuals in late life can be kept alive by *managing* multiple pathologies, but it has not made substantive improvements to healthy life span.

Unlike inoculating against smallpox or the provision of sanitation, which added decades to life-expectancy, eliminating all (200+) types of cancer is estimated to only increase life-expectancy at birth in the USA by approximately three years.^12^ That number is lower than most people expect because they fail to realize that most people who now die of cancer are over the age of 60, and being immune to cancer does not make one immune to the other diseases of late-life, such as heart disease or stroke.

Ageing populations, coupled with the reality of co-morbidity in late life, means we must expand the cognitive toolbox beyond the disease-specific approach to human health if we hope to increase the opportunities for health in late life. A systems biology approach which studies the interactions between the components of a biological system must be adopted if we hope to add health to late life.^13^ This means that interdisciplinary methodologies must be encouraged and developed, and new organizational linkages must be pursued that transcend the current constraints of the disease-specific approach to health research. The IPB would help ensure we expand the cognitive toolbox in new and useful ways to address the unique health challenges of the 21st century. By doing so we may be able to confer enormous health benefits upon the two billion people worldwide who are expected to be over the age of 60 by the middle of this century.

## Conclusion

The IPB would be an interdisciplinary institute, bringing together researchers from the natural and social sciences. The central goal of the institute would be to translate basic scientific research on exemplar positive phenotypes into safe and effective clinical and environmental interventions that could promote human health and happiness. A strategic focus on exemplar positive phenotypes would bring to the fore not only research on exceptional longevity, but also play, resilience, happiness and high cognitive ability.

Celebrating and supporting scientific research into exemplar positive phenotypes, by designing and funding a specific institute dedicated to positive biology, would help legitimize many important areas of scientific research – from longevity science and positive psychology to research into high cognitive functioning and play. These fields of research have tended to be viewed as, at best, ‘intellectual curiosities’ and, at worst, a waste of public funding. By creating the IPB, the NIH would be sending a clear message that the study of exemplar phenotypes is an intricate part of well-ordered science and medicine for the 21st century. The IPB has the potential to be a ‘game changer’ in research on human health and wellbeing. And for that reason it is deserving of its own institute, alongside the NIH's institutes of disease.

## DECLARATIONS

### Competing interests

None declared

### Funding

None

### Ethical Approval

Not applicable

### Guarantor

CF

### Contributorship

CF is the sole author

### Acknowledgements

I am grateful to Bruce Carnes for his helpful comments on an earlier version of this paper
